# Pembrolizumab-Induced Hypophysitis: A Rare Immune-Related Adverse Event in a Patient With Metastatic Non-small Cell Lung Cancer

**DOI:** 10.7759/cureus.82701

**Published:** 2025-04-21

**Authors:** Ammar Al Heyasat, Maida S Chaudhry, Murad Alkharabsheh, Mohammad Bani Amer, Indira Poojary

**Affiliations:** 1 Department of Internal Medicine, Crestwood Medical Center, Huntsville, USA; 2 Department of Internal Medicine, DHR Health Institute for Research and Development, Edinburg, USA

**Keywords:** adrenal insufficiency, hypophysitis, immune checkpoint inhibitors (icis), immune-related adverse events (iraes), non-small cell lung cancer (nsclc), pembrolizumab

## Abstract

Immune checkpoint inhibitors (ICIs), such as pembrolizumab, have revolutionized cancer treatment but are associated with immune-related adverse events (irAEs). Hypophysitis, though rare, is a serious endocrine complication that can lead to life-threatening adrenal insufficiency if not promptly recognized and treated.

A 62-year-old man with metastatic non-small cell lung cancer presented with fatigue, headache, and hyponatremia after four cycles of pembrolizumab. Laboratory evaluation revealed secondary adrenal insufficiency (low cortisol and adrenocorticotropic hormone) and central hypothyroidism (low free thyroxine, T4, with inappropriately normal thyroid-stimulating hormone). Magnetic resonance imaging of the brain showed a normal pituitary gland. A diagnosis of pembrolizumab-induced hypophysitis was made based on clinical and biochemical findings. Pembrolizumab was temporarily withheld, and the patient was started on hydrocortisone and levothyroxine replacement. Symptoms improved within one week, and pembrolizumab was resumed without complications.

This case highlights the importance of monitoring for endocrine irAEs in patients receiving ICIs, even without radiographic abnormalities. Early recognition and treatment are critical to reduce morbidity. Clinicians should suspect hypophysitis in patients presenting with nonspecific symptoms such as fatigue, headache, or hyponatremia during ICI therapy.

## Introduction

Immune checkpoint inhibitors (ICIs), including pembrolizumab, are a cornerstone of treatment for advanced malignancies such as non-small cell lung cancer (NSCLC) [1]. However, their use is associated with immune-related adverse events (irAEs), which can affect multiple organ systems [2]. Hypophysitis, an inflammation of the pituitary gland, is a rare but potentially life-threatening irAE that may lead to pituitary hormone deficiencies [3]. We present a case of pembrolizumab-induced hypophysitis in a patient with metastatic NSCLC, emphasizing diagnostic challenges and management strategies in the absence of radiographic abnormalities.

## Case presentation

A 62-year-old man with a metastatic NSCLC presented with fatigue, headache, and nausea approximately two weeks after receiving his fourth cycle of pembrolizumab (200 mg administered every three weeks). Physical examination revealed mild dehydration. Laboratory evaluation demonstrated hyponatremia (sodium: 126 mmol/L), low morning cortisol (2.1 μg/dL), and low adrenocorticotropic hormone (ACTH; 5 pg/mL). Thyroid studies revealed a low free T4 level (0.6 ng/dL) with an inappropriately normal thyroid-stimulating hormone (TSH) level (1.2 μIU/mL), consistent with central hypothyroidism.

**Figure 1:**
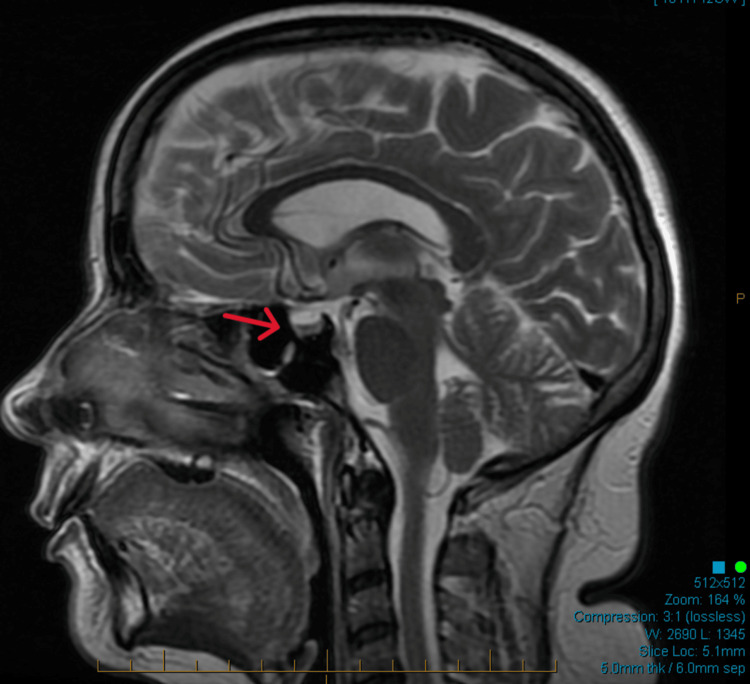
A sagittal brain MRI T2-weighted image showing a normal pituitary gland, which suggests that the gland has the expected size, shape, and signal intensity without abnormalities such as masses or cysts (red arrow) MRI: magnetic resonance imaging

**Figure 2:**
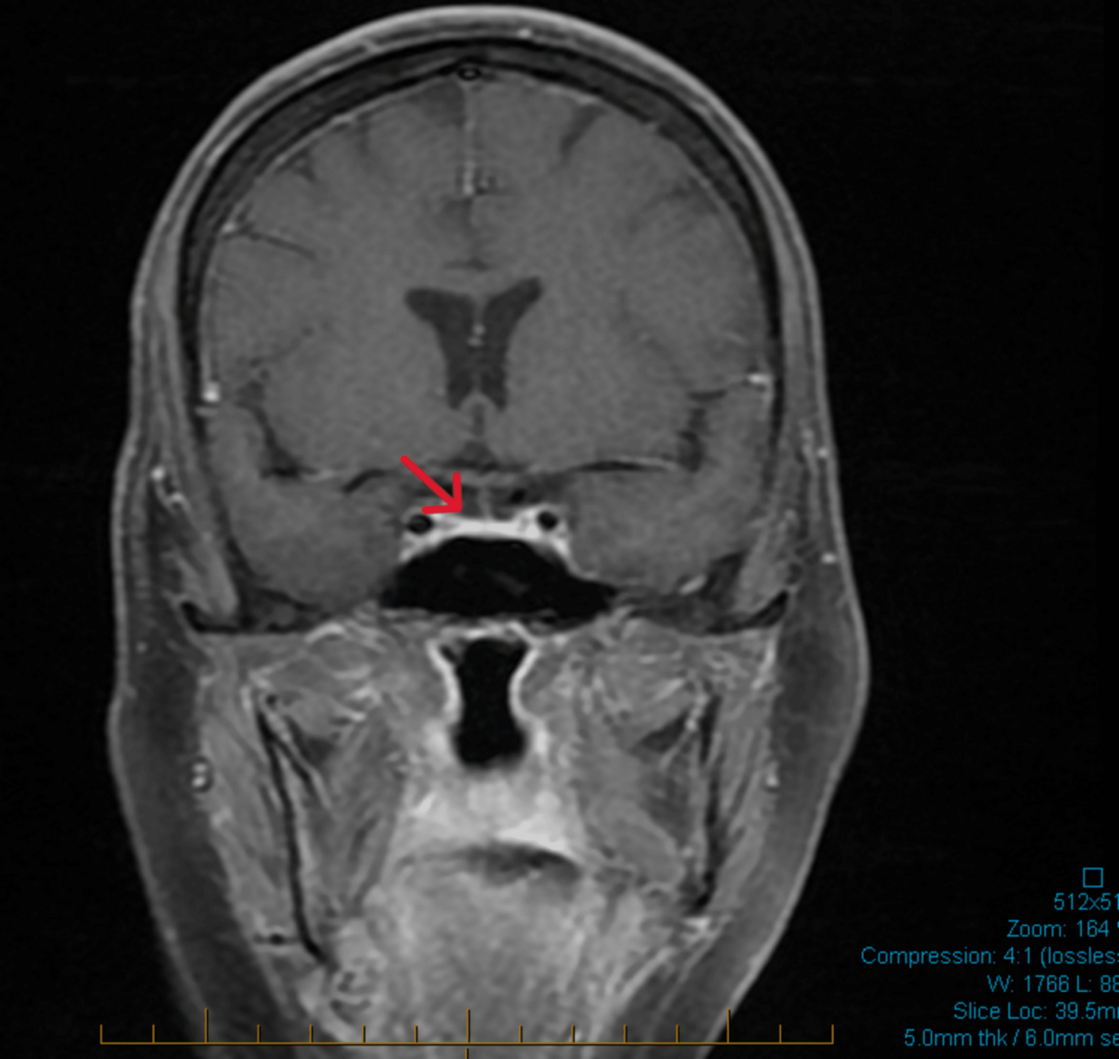
A coronal brain MRI T1-weighted image showing a normal pituitary gland, which suggests that the gland has a typical size, shape, and signal intensity without evidence of structural abnormalities (red arrow) MRI: magnetic resonance imaging

An ACTH stimulation test revealed a suboptimal response (30-minute cortisol: 8 μg/dL; 60-minute cortisol: 10 μg/dL). Brain magnetic resonance imaging (MRI) showed a normal pituitary gland with no structural abnormalities (Figure 1). A comprehensive anterior pituitary panel was obtained, including prolactin, luteinizing hormone, follicle-stimulating hormone, and insulin-like growth factor-1, all of which were within normal limits. Further coronal views confirmed the normal pituitary morphology (Figure 2). These findings supported the diagnosis of pembrolizumab-induced hypophysitis (Table 1), primarily affecting the corticotropic and thyrotropic axes.

**Table 1:** Laboratory findings in pembrolizumab-induced hypophysitis ACTH: adrenocorticotropic hormone; TSH: thyroid-stimulating hormone; LH: luteinizing hormone; FSH: follicle-stimulating hormone; IGF-1: insulin-like growth factor-1; IU: international units

Parameter	Patient value	Reference range	Interpretation
Sodium	126 mmol/L	135-145 mmol/L	Hyponatremia
Potassium	4.2 mmol/L	3.5-5 mmol/L	Normal
Cortisol (AM)	2.1 μg/dL	5-25 μg/dL	Low (adrenal insufficiency)
ACTH	5 pg/mL	10-60 pg/mL	Low
TSH	1.2 μIU/mL	0.4-4 μIU/mL	Inappropriately normal
Free T4	0.6 ng/dL	0.8-1.8 ng/dL	Low (central hypothyroidism)
Prolactin	8 ng/mL	4-23 ng/mL	Normal
LH	4.5 mIU/mL	1.5-9.3 mIU/mL	Normal
FSH	5.2 mIU/mL	1.4-18.1 mIU/mL	Normal
IGF-1	112 ng/mL	52-328 ng/mL (age-adjusted)	Normal
ACTH stimulation test			
Baseline cortisol	2.1 μg/dL	5-25 μg/dL	Low
30-minute cortisol	8 μg/dL	≥18 μg/dL	Suboptimal response
60-minute cortisol	10 μg/dL	≥18 μg/dL	Suboptimal response

Pembrolizumab was withheld, and the patient was started on hydrocortisone 20 mg/day and levothyroxine 75 μg/day. Symptoms resolved within one week, and pembrolizumab was resumed two weeks after symptom resolution, following clinical stabilization on hormone replacement therapy.

The patient was followed in the outpatient setting by endocrinology, with serial hormone monitoring (Table 2) at one, three, and six months after discharge. Cortisol, ACTH, TSH, and free T4 levels remained stable throughout the follow-up period, and the patient experienced no recurrence of hypophysitis or symptoms during six months of ongoing immunotherapy.

**Table 2:** Follow-up endocrine labs after hormone replacement therapy ACTH: adrenocorticotropic hormone; TSH: thyroid-stimulating hormone

Time point	Cortisol (μg/dL)	ACTH (pg/mL)	TSH (μIU/mL)	Free T4 (ng/dL)
One month	6.5	12	1.4	1.1
Three months	7.1	15	1.6	1.2
Six months	6.8	14	1.5	1.2

## Discussion

Hypophysitis is a rare irAE associated with ICIs, particularly anti-programmed death (PD)-1 agents like pembrolizumab. The mechanism involves immune-mediated inflammation of the pituitary gland, often leading to deficiencies in anterior pituitary hormones. While more common with cytotoxic T-lymphocyte-associated antigen 4 inhibitors, pembrolizumab-induced hypophysitis is increasingly recognized, with presentations ranging from isolated ACTH deficiency to full hypopituitarism [4-6]. This condition can occur in the absence of radiologic findings, which emphasizes the importance of biochemical screening even when imaging is unremarkable [4,7].

Our patient developed adrenal insufficiency and central hypothyroidism, while the remaining pituitary hormones remained within normal limits. This clinical presentation is consistent with patterns observed in other pembrolizumab-associated cases, where the corticotropic and thyrotropic axes are most commonly affected [4-6,8]. Pituitary MRI is often normal in such cases, as seen here, and should not be relied upon solely to exclude hypophysitis [4,9].

The pathogenesis may involve cross-reactivity to pituitary antigens or immune activation against PD-1-expressing pituitary cells [9,10]. Recent studies suggest that these effects may occur late during treatment, with variable onset, and may persist even after cessation of therapy [6,11].

Hormone replacement with hydrocortisone and levothyroxine remains the cornerstone of management. High-dose steroids are typically reserved for mass effect or acute inflammation, which was not seen here. Pembrolizumab was safely resumed two weeks after symptom resolution and hormone initiation, consistent with other reports suggesting ICIs may be resumed after clinical stabilization [5,7,12].

This case highlights the need for regular endocrine monitoring, even in asymptomatic patients receiving ICIs. Multidisciplinary coordination with endocrinology is critical for optimizing outcomes and long-term management. Serial hormonal evaluations at one, three, and six months after treatment confirmed the biochemical stability in our patient.

## Conclusions

Pembrolizumab-induced hypophysitis requires prompt recognition and hormone replacement to prevent adrenal crisis. Clinicians must maintain a high index of suspicion for endocrine irAEs, even with normal imaging.
